# The Dynamic Oval Pupil

**DOI:** 10.3389/fneur.2019.00075

**Published:** 2019-02-07

**Authors:** Fion D. Bremner, Allan J. Drapkin

**Affiliations:** ^1^Department of Neuro-Ophthalmology, National Hospital for Neurology and Neurosurgery, London, United Kingdom; ^2^Department of Surgery (Neurosurgery), Jersey Shore University Medical Center, Neptune, NJ, United States

**Keywords:** corectopia, oval pupil, midbrain, autonomic function, upgaze paresis

## Abstract

The dynamic oval pupil is defined and its distinction from corectopia, as well as their different clinical significance is proposed. A literature search for instances presenting this condition yielded only 20 such cases with enough clinical data. A review of these cases allows us to draw some tentative conclusions regarding the most likely anatomical location for its causative lesion and the pathophysiological mechanism responsible for its occurrence.

## Introduction

Corectopia, defined as the displacement of the center of the pupil away from the center of the cornea, is usually encountered within the realm of Ophthalmology. It relates mostly to conditions that could be considered “static” because these are anatomically fixed pupillary deformities resulting from congenital (1, 2) or acquired conditions [anterior or posterior synechiae, iris coloboma or sectoral hypoplasia, acute angle closure glaucoma, inflammation, iris ischemia or rubeosis, trauma or post-surgical sequelae, diabetes mellitus (3) among others (4–6)]. Although *corectopia* and *oval pupil* have been usually employed, in the pertinent literature, as synonyms, we propose that these should be distinguished as two quite different conditions. What is understood as an oval pupil in the neurological arena, usually presents itself in the midst of a severe neurological event causing midbrain-tectal dysfunction (7, 8) and it is a *dynamic* alteration of the round shape of the pupil that can occur intermittently, can change its size and/or the orientation of its major axis or return to its normal round shape while being observed, this together with varying effects on its reactivity (7, 9).

This type of dynamic oval pupil has been considered a transitional state preceding the mydriatic and fixed pupil of a full-blown transtentorial herniation (8), and consequently, when detected, should raise the alert of the clinician to act on an urgent basis.

Since the 1970's, with the advent of computed tomography (CT) and later followed by magnetic resonance imaging (MRI), a significant decline in the interest and in the time devoted to a detailed neurological examination has become unfortunately evident in daily practice. Nevertheless, in certain clinical situations, where the neurological status of the patient is unstable, intensive clinical neuromonitoring remains indispensable, because current imaging technology cannot provide the required minute-to-minute assessment, it is expensive and it is not always available. It is in that setting that the detection of a dynamic oval pupil finds its significance.

## Materials and Methods

A careful search of the literature for the headings of “*midbrain corectopia,” “pupillary ectopia,”* and “*oval pupil*”' was carried out. It yielded 10 reports conforming to our definition of “*Dynamic oval pupil*” (7–16). A total of 95 cases were collected from these reports, but their perusal disclosed only 20 cases that provided a minimum of clinical data that justified their inclusion on this study. Their clinical characteristics were then tabulated in order to bring out their commonality (Table 1).

**Table 1:** Summary of 20 previously published cases of the dynamic oval pupil.

**Author, Case**	**Diagnosis**	**Oval pupil**	**Mentation**	**Main neurological deficits**	**Skew deviation?**	**Anisocoria?**	**Course**
Aikawa, #1	Basilar thrombosis	Bilateral	Stupor	VGP+CP+bilateral PT+LRA+CRA	Yes	L>R	Improved
Aikawa, #2	Vertebro-basilar embolus	Bilateral	Stupor	VGP+CP+right PT+LRA,+left LRS+hemiparesis	Yes	R>L	Death
Aikawa, #3	Vertebro-basilar embolus	Bilateral	Awake	VGP+CP+ bilateral LRS+right PT+left cerebellar dysfunction	Yes	R>L	Improved
Aikawa, #4	Vertebro-basilar ischaemia	Bilateral	Stupor	VGP+CP+left PT+right LRS+left LRA+hemiparesis	Yes	R>L	Improved
Aikawa, #5	Vertebro-basilar ischaemia	Bilateral	Somnolence	VGP+CP+ right PT+LRS+left cerebellar dysfunction	Yes	R>L	Improved
Aikawa, #6	Vertebro-basilar ischaemia	Bilateral	Stupor	VGP+quadriparesis +LRA bilaterally	Yes	R>L	Improved
Aikawa, #7	Midbrain infarct	Bilateral	Stupor	Partial ophthalmoplegia+aphasia+right hemiparesis+bilateral LRA	Yes	R>L	Improved
Wilson, #1	Brainstem glioma	Bilateral	Awake	VGP+right hemiplegia+bilateral VI nerve weakness+LRS	No	R>L	Death
Wilson, #2	III ventricle colloid cyst	Bilateral	Coma	VGP+CP+bilateral LRS+PT+divergent strabismus	No	L>R	Death
Wilson, #3	Multiple sclerosis	Bilateral	Awake	Right PT+exotropia+LRS+quadriparesis	No	R>L	Improved
Terao, #1	Brainstem hemorrhage	Left	Coma	Bilateral LRA+CRA	No	R>L	Death
Terao, #2	Hypertensive thalamic bleed	Right	Coma	Right hemiplegia+bilateral LRA+CRA	No	None	Death
Terao, #11	Left thalamic bleed	Bilateral	Coma	Divergent strabismus	Yes	?	Death
Chu/Sadun	Left sphenoid dural AV fistula	Bilateral	Awake	Bilateral mydriasis and hyporeflexia+bilateral LRS	No	L>R	Alive
Lindbauer	Germ cell pineal tumor	Bilateral	Awake	VGP+convergence retraction nystagmus+Collier sign	No	None	Alive
Selhorst	Midbrain infarct	Bilateral	Stupor to coma	VGP+bilateral LRA+Homonymous hemianopsia	No	L>R	Death
Jeret, #1	Brainstem ischaemia	Left	Coma	Bilateral Babinski+LRA	No	L>R	Improved
Jeret, #2	Hypoglycaemia	Right	Coma	Bilateral decerebration+Babinskis	No	None	Improved
Cohen, #1	Periaqueductal bleed	Bilateral	Coma	No response to caloric test+bilateral LRA+CRA	No	None	Death
Cohen, #2	Head trauma	Left	Coma	Decerebration+left LRA+right LRS	No	L>R	Right hemiplegia

*VGP, Vertical gaze paresis (it is not always clear from description as to whether this was supranuclear or infranuclear); CP, Convergence paresis; PT, Ptosis; LRA, Light reflex absent; LRS, Light reflex sluggish; CRA, Consensual reflex absent*.

## Results

Of the cases reviewed, 15 of these were male and 5 were female. There was a similar range of ages for cases caused by vascular insufficiency (30–86) and intra-axial hemorrhage (53–79) but cases caused by tumor were significantly younger (13-42) The state of consciousness varied between stupor and coma in 14 of the 20 cases, somnolence in one (10) and was not altered in five (10, 11, 13, 16). Of those five, the cause was tumoral in two (13, 16), multiple sclerosis in one (16), an AV fistula compressing the midbrain in another (11) and in the fifth one it was due to a midbrain embolus (10).

Anisocoria, when it was recorded, was present in 15 cases, in another case pupillary size varied independently in the two eyes (7), and pupils were equal in size in the remaining four cases [the etiology in these four cases was a thalamic bleed in one (15), a prepontine extra-axial tumor in another (16) a pineal tumor in a third (13) and hypoglycemia in the fourth one (12)].

The pupil light reflex was absent in at least one eye in ten cases, sluggish in six and no information was available in the rest (for each of these cases we have considered the less reactive of the two pupils). A “dynamic oval pupil” was present in all 20 cases: it was bilateral in 15 and unilateral in five.

The most common oculomotor defect associated with the dynamic oval pupil consisted of limited vertical eye movements, particularly upward gaze, which was found in ten cases (9, 10, 13, 16); in six of these cases the Doll's head maneuver did not elicit reflex movement of the eyes in the vertical plane, implying a nuclear or infranuclear deficit (7, 10) but in the remaining cases the assumption is that the deficit was supranuclear. In addition skew deviation was noted in eight cases (10, 15), convergence insufficiency in seven cases (10, 13, 16) and ptosis was present in seven cases [unilateral in five (10, 16) and bilateral in two (10, 16)].

## Discussion

While a dynamic oval pupil is and should be considered an ominous prognostic sign because it represents rostral midbrain dysfunction, a significant difficulty in reviewing potential cases of this condition resides in the detailed clinical observation required not only to detect and record an oval pupil *per se*, but also to include in it that pupil's behavior in time, its reactivity and associated ocular motor deficits. Such information is indispensable to enable the differentiation between a dynamic oval pupil, the object of this work, and a static one or corectopia. Proof of this difficulty is the fact that from 95 cases of oval pupils collected from the literature, only 20 provided a minimum of clinical data sufficient to be able to include these in this study (Table 1).

Perusal of these data revealed that a dynamic oval pupil due to (a) rostral midbrain ischemia (10, 12, 17), (b) multiple sclerosis (16), (c) treatable pineal tumors (13), or (d) hypoglycemia (12) have a relatively benign prognosis, provided the underlying cause responds to treatment, while cases presenting with a dynamic oval pupil secondary to an hypertensive intracranial bleed or a subarachnoid hemorrhage associated with intracerebral hematomata and/or vasospasm have a dismal prognosis due to either transtentorial or tonsillar herniation or intra-axial brainstem hemorrhage (14, 15).

Head trauma, which has also been associated with the occurrence of a dynamic oval pupil secondary to either brain contusion or intracranial hematomata (with or without increased intracranial pressure) (7, 8), constitute an intermediate group. Timely control of the intracranial hypertension, when present, either by medical or surgical means, may improve the condition and revert the oval pupil to its normal shape and reactivity. Only a minority of cases within our review have presented with a dynamic oval pupil without an associated altered state of consciousness. One of these was due to a brainstem embolic event (10), a second case occurred in the course of multiple sclerosis (16) while the other two were tumoral [a germ-cell pineal tumor in one (13) and a brainstem glioma in the other (16)]. In keeping with what has been stated above, it would appear likely that in these cases the oval pupil was the result of a more circumscribed mesencephalic lesion than the ones already described (18–22), not significant enough to cause unconsciousness but sufficient to alter the rostral midbrain function, leading to a dynamic oval pupil, that eventually can resolve.

At this point it would appear worthwhile to analyze the possible physiopathological mechanisms responsible for the occurrence of a dynamic oval pupil.

Since in the overwhelming majority of the reported cases a dynamic oval pupil occurs in association with a severe impairment of consciousness, a fair assumption can be made that the responsible pathology resulting in a dynamic oval pupil affects, in major or minor degree, the ascending reticular activating system (ARAS).

The pupillary light reflex circuit starts at the retinal ganglion cells. Their axons run with the optic nerve, partially decussating at the optic chiasm and then following the optic tract, passing through the lateral geniculate body to reach the pretectal nuclei (mostly the olivary pretectal nuclei) in the rostral midbrain. There they partially decussate through the posterior commissure and reach the Edinger Westphal nuclei where they synapse with the dendrites of preganglionic parasympathetic neurons (E-W_pg_) bringing to them an excitatory influence (23, 24). In addition to this retinal input, neurons from the central mesencephalic reticular formation (cMRF) have also been shown to densely project to the E-W_pg_ nuclei, where they make numerous synaptic contacts of an apparent inhibitory nature (25–27) suggesting that it could play a role in pupillary constriction, vergence and/or accommodation. From the E-W_pg_ nuclei the efferent parasympathetic nerve fibers go, via the oculomotor nerve, into the orbit and synapse at the ciliary ganglion, just behind the globe (28). The axons of these post-ganglionic neurons travel forward in the short posterior ciliary nerves within the suprachoroidal space, to reach the iris (29). Each post-ganglionic fiber innervates about 15–20% of the circumference of the pupillary sphincter muscle, but it also seems to innervate fibers of the opposing dilator pupillary muscle within the same iridic sector (30). The precise function of this dual innervation has not yet been established, but it is thought that this parasympathetic input into a sympathetic effector may cause relaxation of that sector of the dilator fibers, at the same time that fibers of the pupillary sphincter are constricting.

Based on that map, a possible location for the lesion responsible for producing a dynamic oval pupil might be in the area of the cMRF. Such a lesion (hemorrhage, ischemia, tumor, etc.) would result in the severe impairment of consciousness that frequently accompanies a dynamic oval pupil. Furthermore, based on what appears to be a predominantly *inhibitory* influence by the cMRF over the E-W_pg_ nuclei, any damage to the cMRF is expected to result in *disinhibition* of some of these preganglionic parasympathetic neurons. We know this occurs in sleep, when the pupils become uniformly miosed; in pathological states (e.g., trauma) it is likely that the disturbance to cMRF function would be less uniform, resulting in more random and “patchy” firing of these neurons and therefore causing uneven contraction of the sphincter muscle and distortion of the shape of the pupil. This hypothetical mechanism could explain the ‘dynamic’ behavior shown by the dynamic oval pupil which can be observed to change its size, shape and/or the orientation of its major axis over time. If the pathological process is self-limited, the condition will tend to stabilize and potentially improve, with partial or complete normalization of the pupillary shape and reactivity, a clinical course that has been previously reported (10, 16). If on the contrary the condition worsens, and corrective measures are not taken or are not feasible, additional intracranial factors such as increased intracranial pressure and/or midline shift could provoke transtentorial or tonsillar herniation, with increasing pressure on the midbrain and the third cranial nerve, leading to a fixed and dilated pupil and, in many cases, the demise of the patient (15).^.^

The described ocular motor malfunction that frequently accompanies a dynamic oval pupil, gives further support to the proposed hypothetical anatomical location for the lesion causing that dysfunction (31, 32). Thus, impaired vertical voluntary gaze, particularly upward gaze, place the lesion site at the rostral midbrain, specifically involving the rostral interstitial nucleus of the medial longitudinal fasciculus (riMLF), as well as the posterior commissure, the interstitial nucleus of Cajal and the nucleus of Darkschewitsch, where a unilateral lesion is sufficient to cause it (31, 33).

The present study, while confirming the threatening character of the detection of a dynamic oval pupil and the urgency required in its management, also demonstrates that in certain etiological conditions, the prognosis is not necessarily as grim as previously envisioned.

## Author Contributions

AD contributed with the original conception of the study, reviewed the bibliography about it, and wrote the first draft. FB added some additional ideas, wrote some sections of the manuscript, and provided a critical review of the text. Both authors read the final version of the work and approved its submission. Both authors are accountable for all aspects of the work.

### Conflict of Interest Statement

The authors declare that the research was conducted in the absence of any commercial or financial relationships that could be construed as a potential conflict of interest.
